# “To Keep Us, Help Us”: Age Inclusivity at Lehman College, a City University of New York Senior Campus in the Bronx.

**DOI:** 10.1093/geroni/igaf122.4299

**Published:** 2025-12-31

**Authors:** Theresa Lundy, Jesus Estrada, Justine McGovern

**Affiliations:** Lehman College, Bronx, New York, United States; Lehman College, Bronx, New York, United States; CUNY—Lehman College, Bronx, New York, United States

## Abstract

This paper shares findings from an age inclusivity study conducted at Lehman College, February-April, 2025. Located in the Bronx, Lehman is a commuter campus offering degrees to over 12,000 students. It is a federally designated Hispanic-serving institution. Faculty number over 900 and staff, including leadership, exceed 1,400. Facing mounting pressure to increase enrolment, promote retention, and meet the needs of changing student demographics in the context of political, financial and social change, the timing of this project is urgent. Research aims included developing new knowledge about the experiences of older members of the Lehman community; providing data to promote age inclusive policies and practices; and improving the Lehman experience for older students, staff and faculty. With student, faculty and staff ages exceeding national norms, Lehman presents an opportunity for leadership in age inclusivity in higher education. Moreover, with student populations trending older and retirement ages extending later, improving conditions for older community members seeking to develop skills, knowledge and networks and participate meaningfully in society with gainful employment, is essential. The Age Inclusivity Survey was sent by email to the Lehman community. Participation was anonymous and voluntary. Key findings include lack of awareness about factors that affect older community members and existing policies and practices designed to enhance the Lehman experience for older members. Qualitative comments revealed disappointment and frustration with, and some appreciation for, campus resources. Implications include raising awareness of diverse experiences at Lehman and extending the reach of inclusivity to improve the Lehman experience for all.

